# Correlation between higher lateral tibial slope and inferior long term subjective outcomes following single bundle anterior cruciate ligament reconstruction

**DOI:** 10.1186/s13018-024-04795-9

**Published:** 2024-05-28

**Authors:** Yu-Chieh Hung, Chieh-An Chuang, Shang-Yu Yao, Keng-Yi Lin, Shih-Feng Hung, Yi-Jou Chen, Chih-Hao Chiu, Chin-Shan Ho, Cheng-Pang Yang, Yi-Sheng Chan

**Affiliations:** 1 Department of Orthopedic Surgery, Keelung Chang Gung Memorial Hospital, Keelung City, 204 Taiwan; 2 Comprehensive Sports Medicine Center, Taoyuan Chang Gung Memorial Hospital, Taoyuan City, 333 Taiwan; 3 Department of Orthopedic Surgery, Linkou Chang Gung Memorial Hospital, Taoyuan City, 333 Taiwan; 4 Department of Orthopedic Surgery, Taoyuan Hospital, Ministry of Health and Welfare, Taoyuan City, 333 Taiwan; 5 Graduate Institute of Sports Science, National Taiwan Sport University, Taoyuan City, 333 Taiwan

**Keywords:** Long-term outcome in ACLR, Single bundle anterior cruciate ligament reconstruction, High tibial slope, Lateral tibial slope, Steep tibial slope

## Abstract

**Background:**

The impact of anatomical factors, such as the lateral tibial slope (LTS), on outcomes following anterior cruciate ligament (ACL) reconstruction is an area of growing interest. This study was led by the observation that patients with a higher LTS may have different recovery trajectories.

**Hypothesis/Purpose:**

The purpose of this study was to investigate the correlation between a higher LTS and long term subjective outcomes following single-bundle ACL reconstruction.

**Study Design:**

This study was designed as a retrospective cohort study.

**Methods:**

The study comprised 138 patients who underwent single-bundle ACL reconstruction. The LTS was measured on preoperative radiographs. Patient-reported outcome measures (PROMs) were collected, which included the Lysholm Knee Score, UCLA Activity Score, IKDC Score, and Tegner Activity Score, over a mean follow-up duration of 137 months.

**Results:**

A significant negative correlation was found between LTS and all measured PROMs (*p* < 0.001). The established cut-off value of LTS distinguishing between “Good” and “Fair” Lysholm scores was 8.35 degrees. Female patients have statistically significant higher LTS and lower PROMs scores than male. Patients with LTS greater than or equal to 8.35 had significantly lower PROMs, indicative of poorer functional and subjective outcomes.

**Conclusion:**

Our findings suggest that a higher LTS is associated with inferior subjective outcomes following single-bundle ACL reconstruction in long term. The LTS cut-off value of 8.35 degrees could potentially be used as a reference in preoperative planning and patient counseling.

**Clinical relevance:**

Understanding the relationship between LTS and ACL reconstruction outcomes could inform surgical planning and postoperative management. These findings highlight the need to consider anatomical variances, such as LTS, when assessing patient-specific risks and recovery expectations, contributing to the advancement of personalized care in sports medicine.

## Introduction

The anterior cruciate ligament (ACL) is one of the most commonly injured ligaments in the human knee, with a high incidence among athletes in particular [1–3]. ACL injuries significantly disrupt the kinematics of the knee, often leading to instability that can predispose the joint to secondary injuries, such as meniscal tears and early onset knee osteoarthritis [4, 5]. The role of anterior cruciate ligament reconstruction (ACLR) is pivotal in this context, as it aims to restore knee stability and function [6, 7]. By reconstructing the damaged ligament, ACLR helps in realigning the knee joint, thereby reducing the risk of further intra-articular injuries, and slowing the progression towards degenerative joint diseases [8–10]. Despite ongoing advancements in anatomical understanding, biomechanical insights, and improvements in surgical techniques for anterior cruciate ligament (ACL) reconstruction, the incidence of graft failures persists, with reported rates ranging from 0 to 12.3% [11–13]. 

The field of anterior cruciate ligament reconstruction (ACLR) primarily utilizes two techniques: single-bundle and double-bundle reconstruction [14, 15]. *Current concept of single bundle ACLR focuses on restoring the anatomic ACL by placing the tibial and femoral in their native sites.* [16]. On the other hand, double-bundle ACLR aims to mimic the native ACL anatomy more closely by reconstructing both the anteromedial(AM) and posterolateral(PL) bundles, potentially offering a more anatomically accurate restoration [17–20]. However, some studies have established that there are no clinically significance difference in patient-reported outcome measures(PROMs) between single bundle and double bundle ACLR patients [21–24]. No consensus has been made regarding the superiority of one method over the other [4, 25]. 

Moreover, many studies investigate the association between tibial slope and graft failure rate after ACLR [26–28]. A growing body of research suggests that anatomical characteristics, such as the lateral tibial slope (LTS), may influence both the risk of ACL injury and outcomes after ACL reconstruction [29, 30]. Higher LTS has been associated with an increased risk of ACL injury due to increased anterior tibial translation [31–34], and it also increases the risk of graft failure after reconstruction. Despite the evidence linking LTS to ACL injury risk, less is known about the impact of LTS on subjective patient outcomes following single bundle ACL reconstruction. Additionally, patient-reported outcome measures (PROMs) have demonstrated their effectiveness in distinguishing between favorable and unfavorable outcomes in medical treatments [35]. *Previous research has explored the relationship between lateral tibial slope (LTS) and graft failure following anterior cruciate ligament reconstruction (ACLR), proposing various cutoff points to differentiate between high-risk (graft failure) and low-risk (nonfailure) groups.* [27, 28] *However, these studies often present varying conclusions, partially attributable to the differing methodologies used to measure LTS.* [26].

This study aims to investigate the correlation between a higher LTS and subjective outcomes following single bundle ACL reconstruction. Our hypothesis is that patients with a higher LTS may report inferior long term subjective outcomes after the surgery, potentially due to persistent instability or other factors.

## Materials and methods

### Study design and patient selection

This was a retrospective analysis of 138 patients (138 knees) who had undergone single-bundle anterior cruciate ligament (ACL) reconstruction by a single experienced orthopedic surgeon (YS Chan) in our institution between January 2005 and December 2014. Last follow up was in 2023.

*Patients presenting with multi-ligament injuries, a history of previous knee surgeries, malignant bone tumors, congenital knee anomalies and deformities, or knee malalignment requiring osteotomy were excluded from the study. Additionally, cases involving combined ACLR/LET (lateral extra-articular tenodesis) or ACLR/ALLR (antero-lateral ligament reconstruction), those who had undergone revision surgeries, and individuals lost to follow-up were also not included.*

### Data collection

Demographic data (age, sex, BMI) and clinical characteristics (status of medial and lateral meniscus) were obtained from patient records. The lateral tibial slope (LTS) and medial tibial slope (MTS) were measured preoperatively on lateral knee radiographs by two independent observers.

### Surgical techniques

All surgeries were performed under general anesthesia with standard procedures. Meniscus tears were repaired using either the all-inside, inside-out, or outside-in technique, depending on the location and extent of the tear. Following meniscus repair, hamstring tendons were harvested for use as autografts. The single-bundle ACL reconstruction commenced with precise anatomical placement of the graft to mimic the native ACL’s anteromedial bundle. The goal was to achieve optimal alignment and tensioning, thereby ensuring knee stability and function. The procedure involved drilling a tunnel through the tibia, then navigating up to the femoral footprint of the original ACL. After positioning the graft, fixation was carried out first at the femoral side, typically using an interference screw, and then proceeding with secure fixation within the tibial tunnel. This method is intended to restore knee stability, allow for the regaining of full range of motion, and facilitate a return to pre-injury activity levels after a period of rehabilitation.

### Outcome measures

The mean follow-up time was 137 (ranging from 102 to 221months). The functional and subjective outcomes of patients were evaluated using four well-established instruments: the Lysholm Knee Scoring Scale the University of California Los Angeles (UCLA) Activity Score, the International Knee Documentation Committee (IKDC) Score, and the Tegner Activity Scale. These outcome measures were collected preoperatively and at regular postoperative intervals (3 months, 6 months, 12 months, and then annually). The scores at the final follow-up were used for analysis.

### Radiographic evaluation

**Fig. 1:**
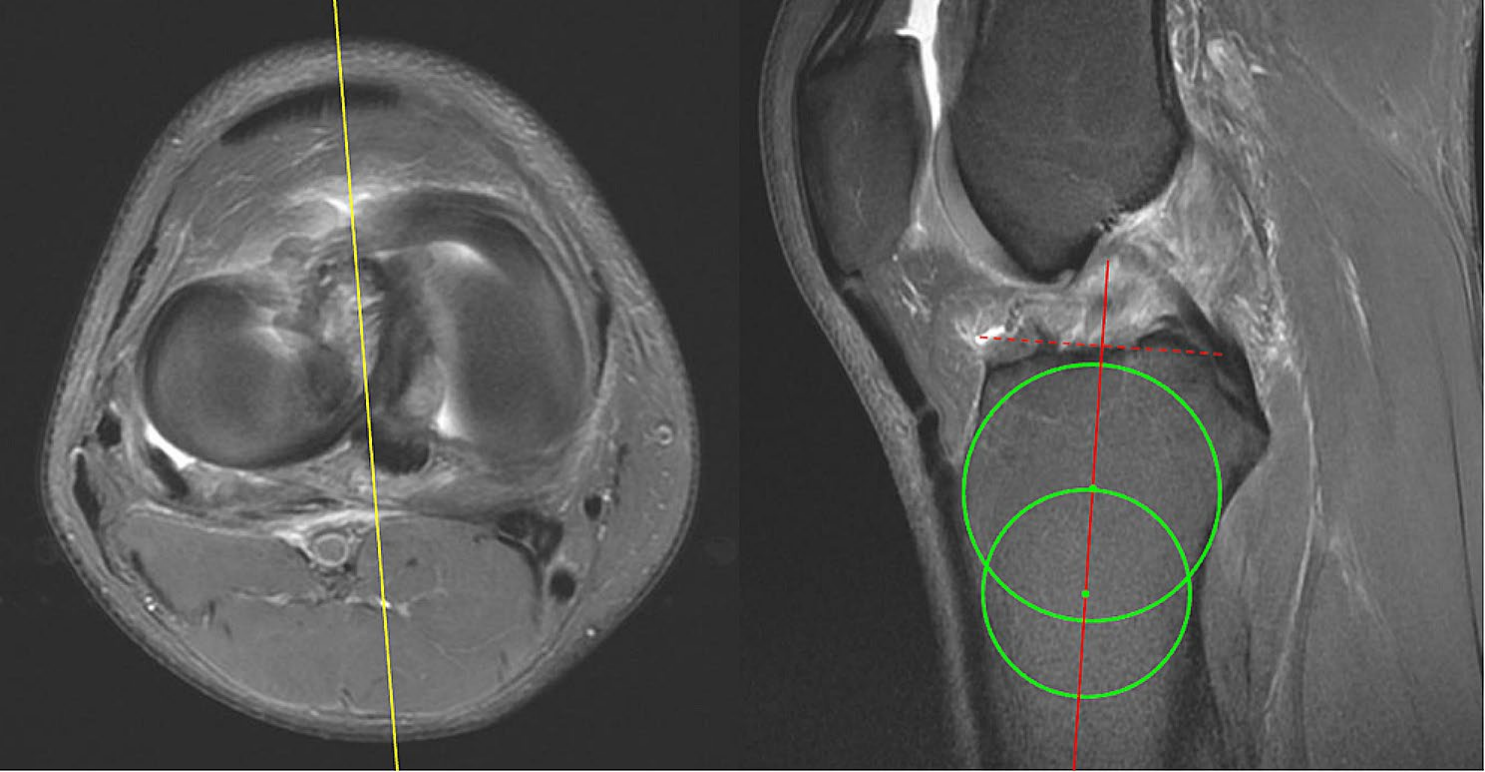
Establishing the longitudinal axis: The reference line (yellow) is adjusted to the center of the tibial plateau. The longitudinal axis (red) is established by connecting the center of the two green circles. The dotted line is defined as being perpendicular to the longitudinal axis

**Fig. 2:**
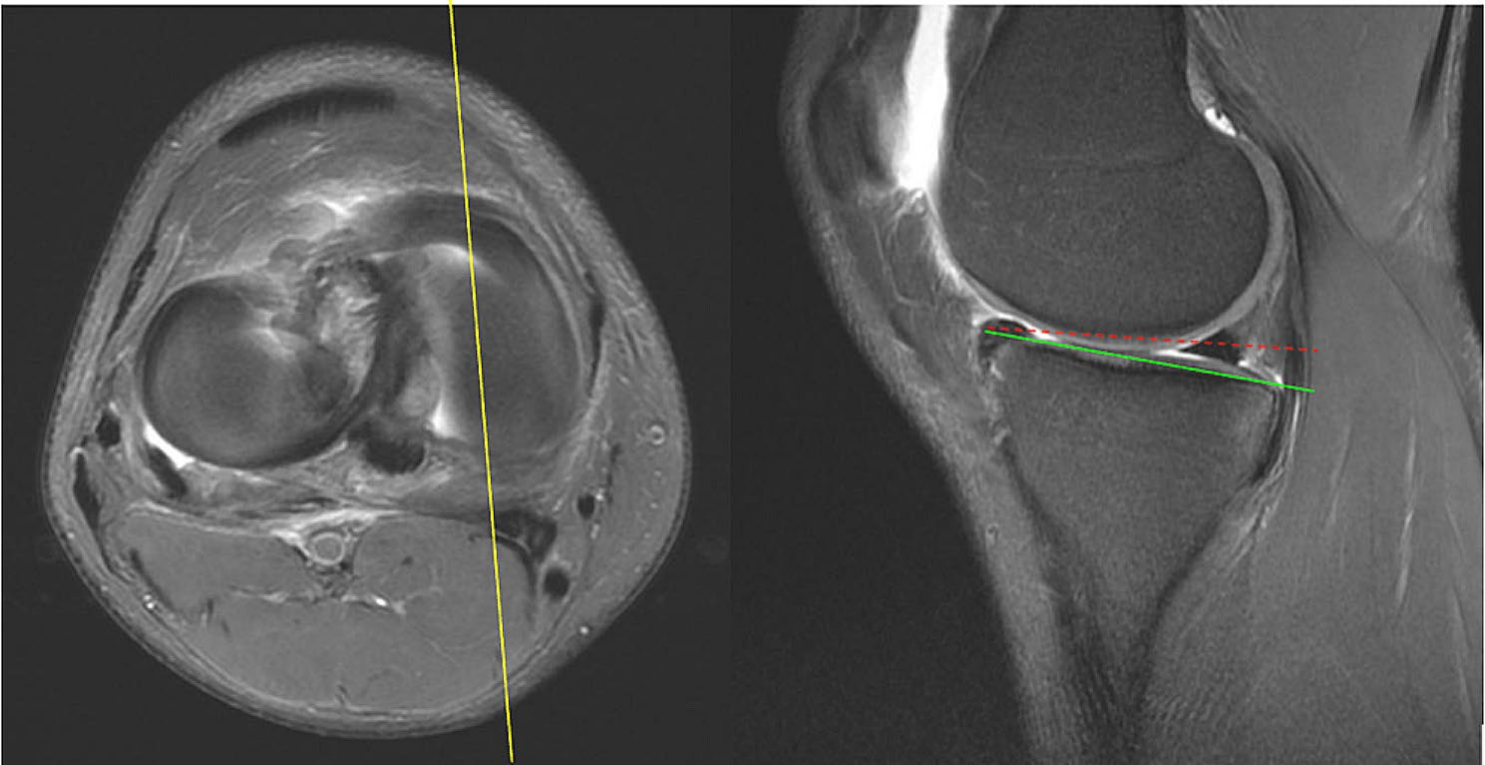
Measuring the medial tibial slope: The reference line is adjusted to the center of the medial tibial plateau. Connect the peak points of the anterior and posterior aspects of the plateau on the Sagittal MRI (green line). The angle between the green line and the dotted red line is defined as the medial tibial slope

**Fig. 3:**
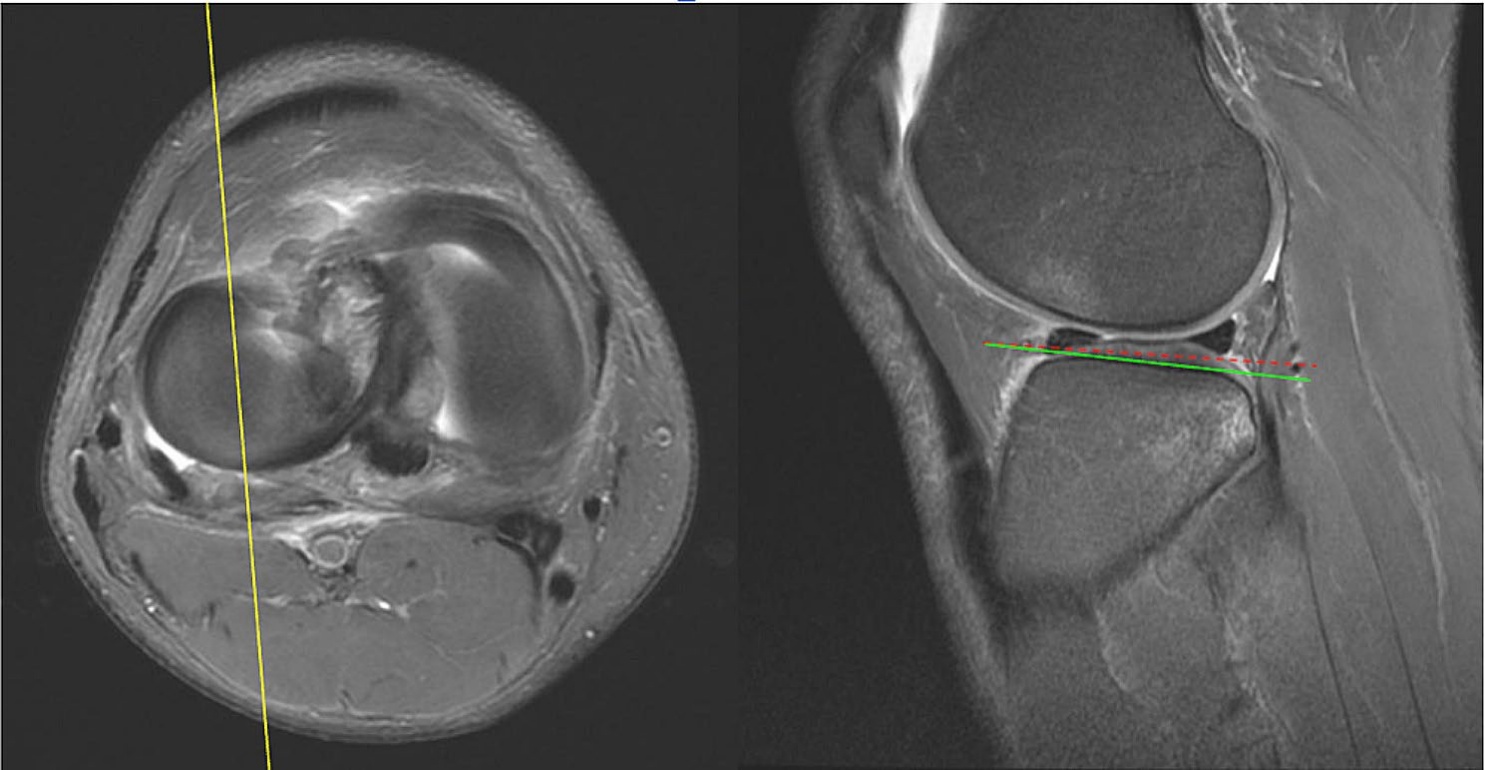
Measuring the lateral tibial slope: The reference line is adjusted to the center of the lateral tibial plateau. Connect the peak points of the anterior and posterior aspects of the plateau on the Sagittal MRI (green line). The angle between the green line and the dotted red line is defined as the lateral tibial slope

Magnetic resonance imaging (MRI) T1 sequence was utilized to assess lower limb alignment and lateral tibial slope (LTS) in our study population. The tibial slopes were measured by two orthopedics residents (YC Hung and KY Lin). The final measurements were confirmed by an orthopedics attending physician (CP Yang). The procedures used to determine the tibial slope were based on the method described by Hashemi et al. and Jahn et al. [36–38] MRI scans were viewed using the Picture Archiving and Communication Systems (PACS). The sagittal image was adjusted until its reference line on the axial view MRI images was at the center of the tibial plateau. Alignment was measured by drawing two circles on the sagittal MRI image of the proximal tibia. The first circle circumscribes the anterior, posterior, and cranial tibial cortex bone. The second circle is adjusted to the anterior and posterior cortical margin with its center on the perimeter of the first. The line that passes through both centers of the circles is defined as the longitudinal axis. Once the axis is established, the sagittal image was determined by scrolling the reference line on the axial images until it is at the center of the medial tibial plateau. A line is drawn connecting the peak points on the anterior and posterior aspects of the plateau. The slope of the lateral tibial plateau was measured as the angle between the line connecting the peak points and the line perpendicular to the longitudinal axis. (Figs. 1, 2 and 3)

### Statistical analysis

We used the Statistical Package for the Social Sciences (IBM SPSS Statistics 23.0) (IBM Inc., Armonk, NY, USA) and Microsoft Office Excel (Microsoft Office 2016). Continuous variables were reported as mean ± standard deviation, and categorical variables were presented as frequencies. Differences between groups for continuous variables were evaluated using the independent samples t-test, while differences for categorical variables were assessed with the Chi-square or Fisher’s exact test as appropriate. Subsequently, the relationship between the LTS and the subjective outcomes (Lysholm Knee Score, UCLA ratings, IKDC, Tegner Activity Score) was assessed using independent t test, Mann-Whitney U test or one-way Analysis of Variance (ANOVA). A p-value of less than 0.05 was considered statistically significant. A receiver operating characteristics (ROC) analysis was performed to establish the optimal cut off LTS value for good and fair Lysholm score.

## Results

### Patient demographics

A total of 138 patients (138 knees) were included in this study. Pre-operation demographic data were as follows. There were 82 males and 56 females. The mean age at surgery was 30.18 ± 10.6. The mean BMI of the patients was 25.01 ± 3.76. The mean lateral tibial slope and medial tibial slope was 7.64 ± 3.29 and 7.71 ± 3.08, respectively. A total of 87 patients had lateral meniscus tear and 56 had medial meniscus tear. (Table 1)

**Table 1:** Demographic parameters

Pre-operation demographic data	
Patient numbers	138
Sex (Male: Female)	82:56
Age (years)	40.82 ± 10.51
BMI (kg/m^2^)	25.01 ± 3.76
Mean LTS	7.63 ± 3.29
Mean MTS	7.71 ± 3.08
**Medial meniscus**	
Intact	82
Non-intact	56
**Lateral meniscus**	
Intact	51
Non-intact	87
Mean F/U duration (Month)	137
Values are mean ± standard deviation. BMI: body mass index LTS: Lateral tibial slope MTS: Medial tibial slope F/U: follow-up	

### Tibial slope and PROMS correlation

**Fig. 4:**
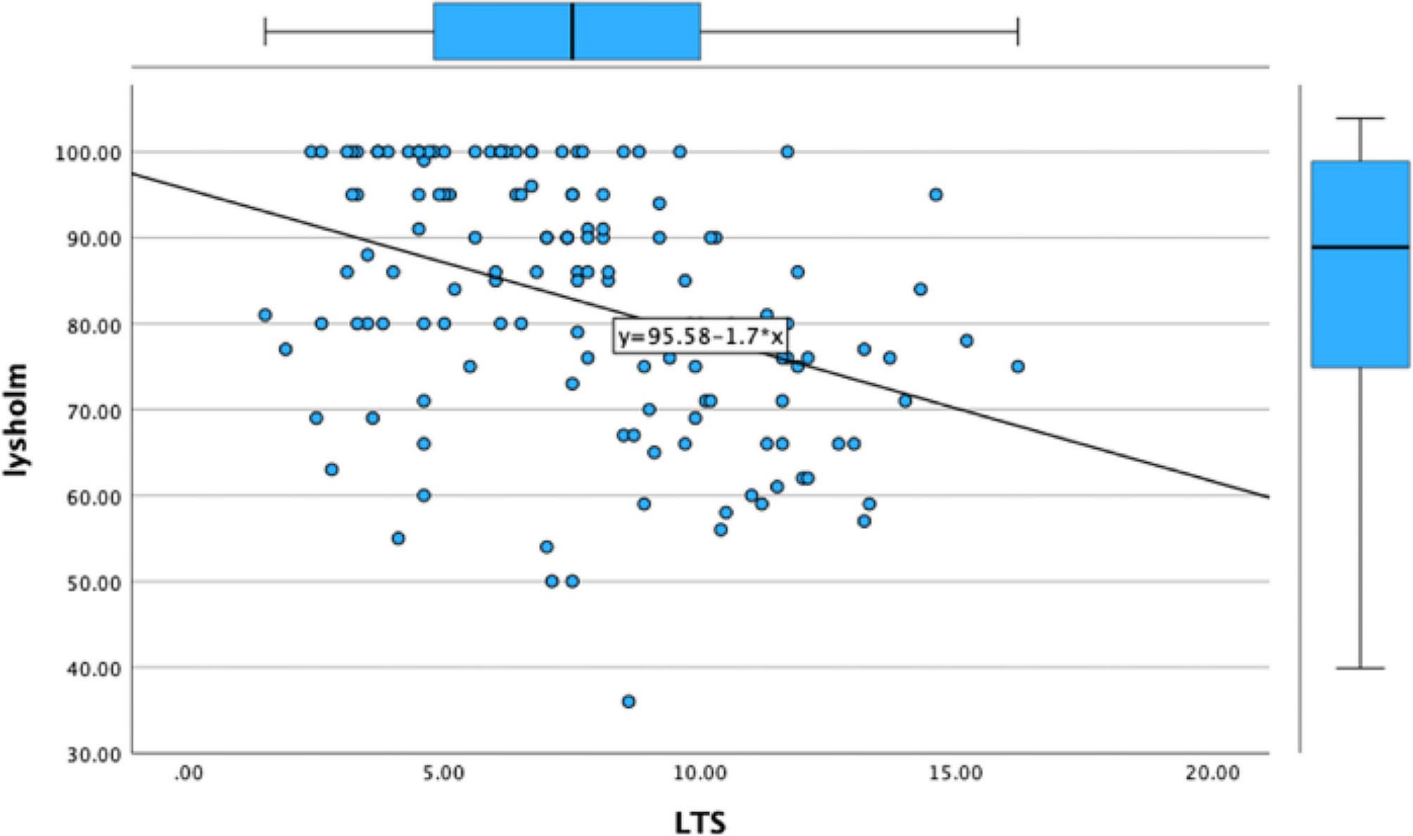
Linear correlation between lysholm score and LTS

**Fig. 5:**
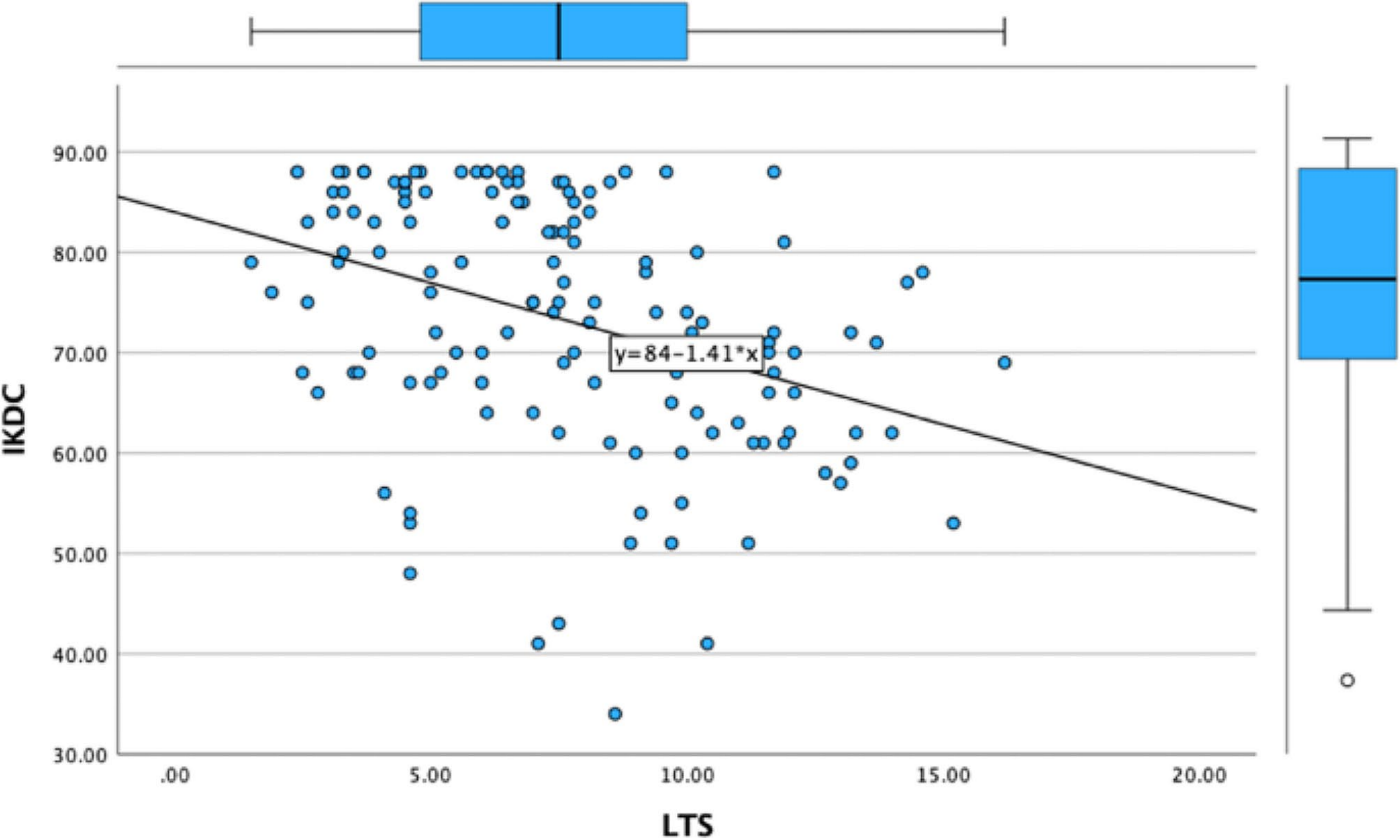
Linear correlation between IKDC score and LTS

**Fig. 6:**
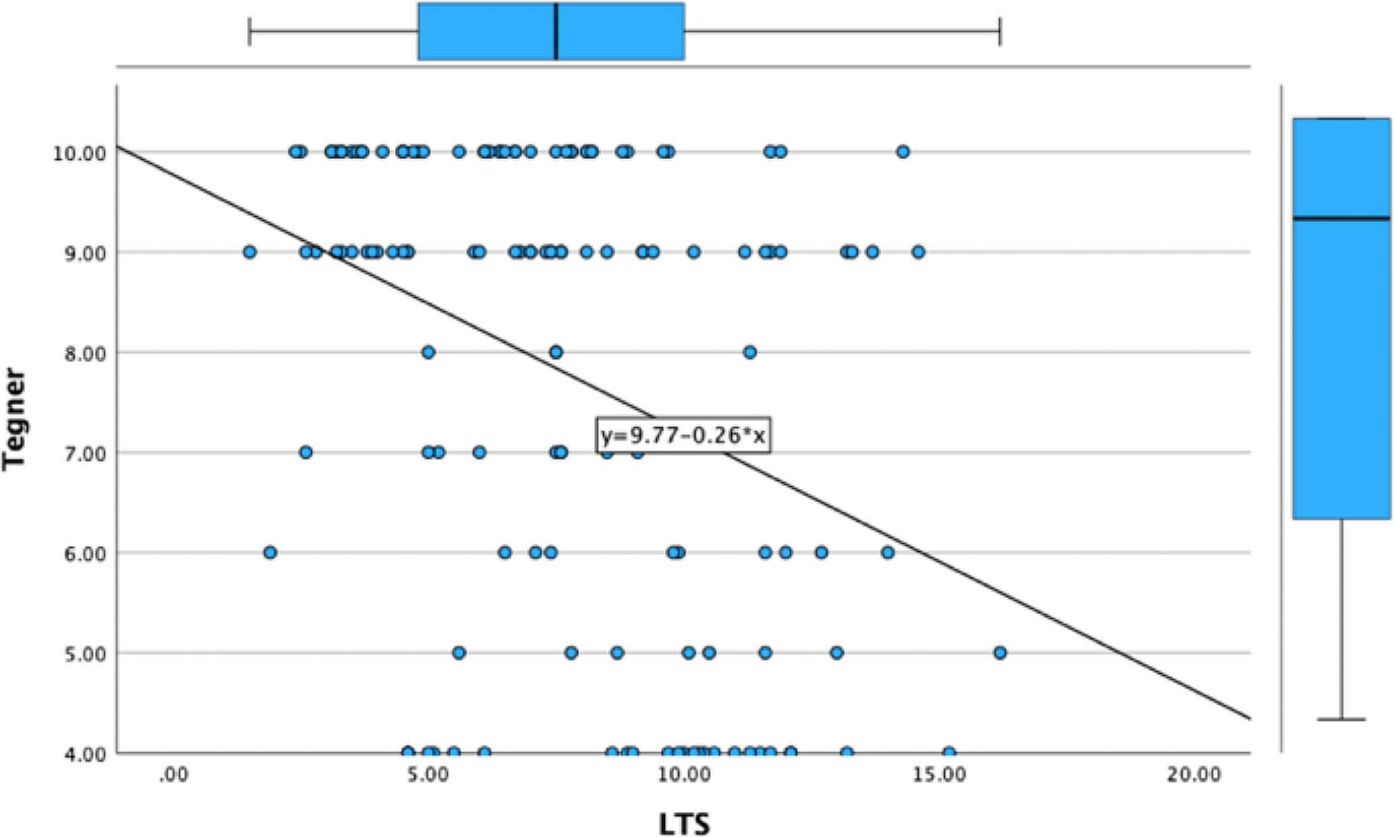
Linear correlation between tegner score and LTS

The lateral tibial slope exhibited a significant negative correlation with postoperative patient-reported outcome measures (PROMs) — including the Lysholm Knee Score(*r*=-0.383, *p* < 0.001), the UCLA Activity Score(*r*=-0.366, *p* < 0.001), the International Knee Documentation Committee (IKDC) (*r*=-0.379 *p* < 0.001)Score, and the Tegner Activity Score (*r*=-0.366, *p* < 0.001) — with a p-value of less than 0.001 for all scores. Meanwhile, the medial tibial slope also displayed a statistically significant negative correlation with these PROMs, although the extent of this correlation was less pronounced compared to that of the lateral tibial slope. The correlation value for Lysholm score was − 0.328 (*p* < 0.001), Tegner score was − 0.300(*P* < 0.001) and IKDC score was − 0.290(*p* = 0.001). The correlation analysis results are listed in Figs. 4, 5 and 6; Table 2.

**Table 2:** Correlation analysis results

	Lysholm score		Tegner score		IKDC score	
	rp	p	rp	p	rp	p
Lateral Tibial Slope	-0.366	0.000	-0.383	0.000	-0.379	0.000
Medial Tibial Slope	-0.300	0.000	-0.328	0.000	-0.290	0.001

### Tibial slope and PROMS comparison of male and female

This study found that the average lateral tibial slope (LTS) in females was 8.41 ± 3.16 degrees, significantly higher than the 7.11 ± 3.28 degrees observed in males (*p* < 0.05). However, the difference in the medial tibial slope (MTS) between genders was not statistically significant, with females averaging 7.98 ± 3.06 degrees and males 7.52 ± 3.09 degrees. When comparing patient-reported outcome measures (PROMs) between male and female patients, there was a significant statistical difference, indicating worse postoperative outcomes for females. Specifically, the Lysholm score averaged 79.20 ± 12.98 in females compared to 84.95 ± 15.20 in males, the IKDC score was 69.80 ± 11.49 in females and 75.56 ± 12.24 in males, and the Tegner score was 7.09 ± 2.50 in females compared to 8.29 ± 2.05 in males. (Table 3.)

**Table 3:** Statistical analysis between female and male

	Female (*n* = 56)	Male (*n* = 82)	
Patient data			
Side, right/left	26/30	46/36	
Medial meniscus lesion, no/yes	35/21	47/35	
Lateral meniscus lesion, no/yes	28/28	23/59	
PCL, intact/nonintact	55/1	82/0	
MCL, intact/nonintact	51/5	79/3	
LTS, degrees	8.41 ± 3.16	7.11 ± 3.28	*p* < 0.05*
MTS, degrees	7.98 ± 3.06	7.52 ± 3.09	*P* = 0.39
Lysholm	79.20 ± 12.98	84.95 ± 15.20	*p* < 0.05*
IKDC	69.80 ± 11.49	75.56 ± 12.24	*p* < 0.05*
Tegner	7.09 ± 2.50	8.29 ± 2.05	*p* < 0.05*
Values are mean ± standard deviation. LTS: Lateral tibial slope MTS: Medial tibial slope Lysholm: Lysholm Knee Scoring Scale IKDC: International Knee Documentation Committee Score Tegner: Tegner Activity Scale *: *p* < 0.05, statistical significance			

### Establishing a cut-off LTS point

**Fig. 7:**
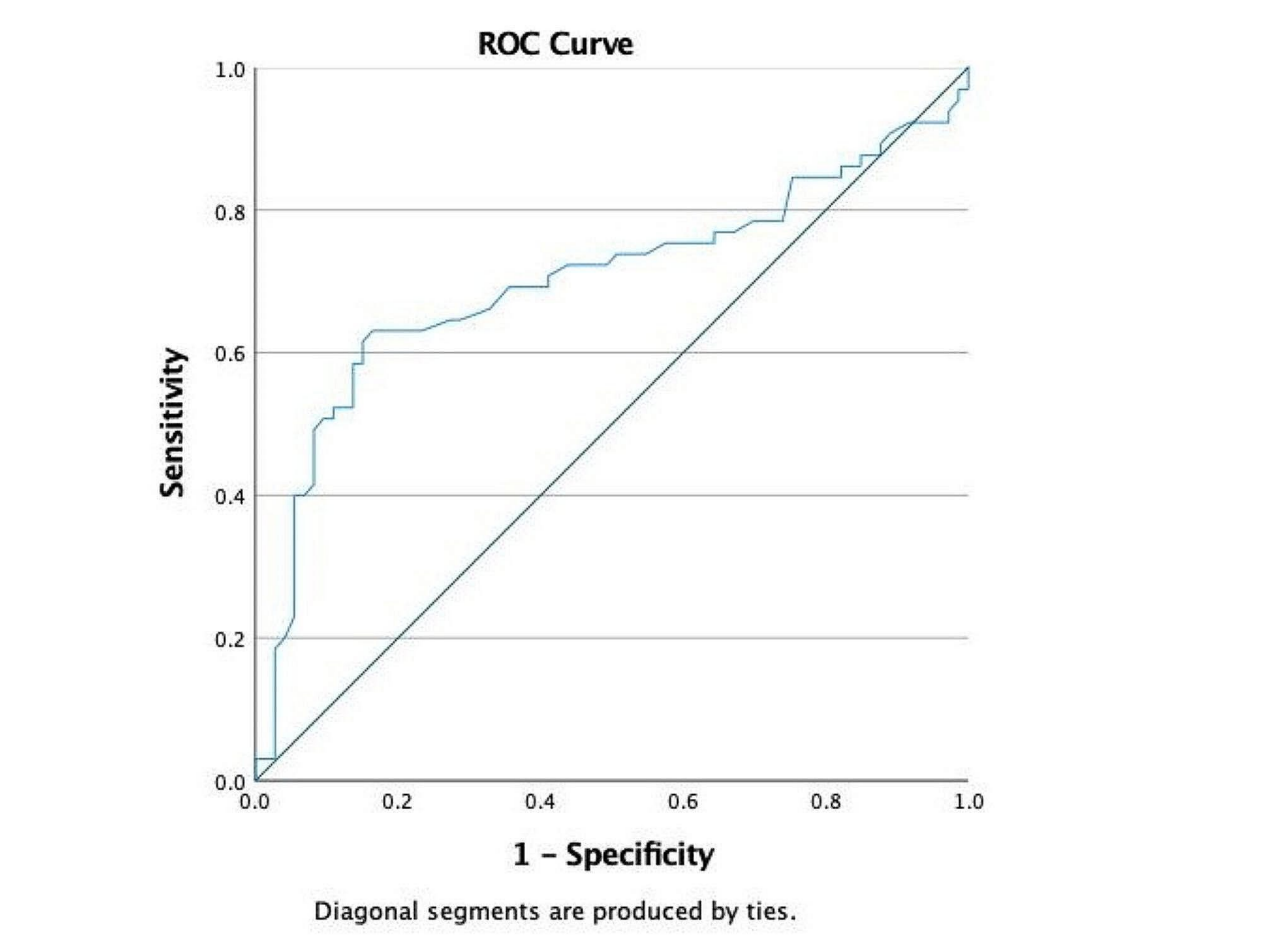
ROC curve analysis

We categorized the patients into four groups, Excellent (95–100), Good (84–94), Fair (65–83) and Poor (< 65), based on the postoperative Lysholm score. Mean LTS in the groups were 5.87 ± 2.43 (*n* = 43), 7.56 ± 2.38(*n* = 30), 8.73 ± 3.77(*n* = 48) and 9.16 ± 3.22(*n* = 17) respectively. (Table 4.) The optimal cut-off LTS between Good and Fair Lysholm score on ROC curve was 8.35. (Fig. 7.)

**Table 4:** Lysholm score rankings

Lysholm	Number	LTS mean (degrees)
**Excellent** **(95–100)**	43	5.87 ± 2.43
**Good** **(84–94)**	30	7.56 ± 2.38
**Fair** **(65–83)**	48	8.73 ± 3.77
**Poor** **(< 65)**	17	9.16 ± 3.22
. One way ANOVA, *p* < 0.001 . LTS cutoff between good and fair: 8.35		

This study then divided the patients based on the cut-off point of LTS as established via ROC curve analysis. This yielded two groups: those with LTS less than 8.35 (*n* = 85) and those with LTS greater than or equal to 8.35 (*n* = 53). Comparatively, patients with a high LTS (greater than or equal to 8.35) had significantly lower scores on all PROMs. Specifically, the Lysholm score was 74.17 ± 13.26 in this group compared to 87.88 ± 12.81 in the group with LTS less than 8.35 (*p* < 0.001). Similarly, the IKDC score was lower in the high LTS group (66.55 ± 11.20 vs. 77.39 ± 10.99, *p* < 0.001). The Tegner Activity Score also followed the same trend (6.55 ± 2.39 vs. 8.59 ± 1.88, *p* < 0.001). (Table 5.)

In summary, patients with a higher lateral tibial slope (LTS ≥ 8.35) experienced significantly inferior subjective and functional outcomes following single-bundle ACL reconstruction as evidenced by their lower scores on the Lysholm Knee Score, UCLA Activity Score, IKDC Score, and Tegner Activity Score.

**Table 5:** Comparison of patient demographic and PROMs between lateral tibial slope < 8.35° and lateral tibial slope ≥ 8.35° groups

	LTS < 8.35 (*n* = 85)	LTS ≥ 8.35 (*n* = 53)	
Patient data			
Age, yr.	30.18 ± 10.61	27.66 ± 10.09	*p < 0.05
Height, cm	167.33 ± 8.80	167.50 ± 9.47	
Weight, kg	70.94 ± 15.38	70.39 ± 15.42	
Side, right/left	41/44	32/21	
Medial meniscus lesion, no/yes	49/36	33/20	
Lateral meniscus lesion, no/yes	32/53	19/34	
PCL, intact/nonintact	85/0	52/1	
MCL, intact/nonintact	80/5	50/3	
MTS, degrees	6.15 ± 2.24	10.21 ± 2.54	
Follow up, yrs.	137.22 ± 31.05	136.70 ± 26.19	
Revision	2	3	
Patient subjective outcome			
Lysholm	87.88 ± 12.81	74.17 ± 13.26	*p < 0.001
UCLA	8.59 ± 1.88	6.55 ± 2.39	*p < 0.001
IKDC	77.39 ± 10.99	66.55 ± 11.20	*p < 0.001
Tegner	8.59 ± 1.88	6.55 ± 2.39	*p < 0.001
(Independent t test) Values are mean ± standard deviation. LTS: Lateral tibial slope Lysholm: Lysholm Knee Scoring Scale IKDC: International Knee Documentation Committee Score Tegner: Tegner Activity Scale *: *p* < 0.05, statistical significance			

## Discussion

This study aimed to investigate the correlation between a higher lateral tibial slope (LTS) and inferior subjective outcomes following single-bundle anterior cruciate ligament (ACL) reconstruction over an extended period. Our findings support the theory that a greater LTS is associated with poorer subjective outcomes post-single-bundle ACL reconstruction in the long term. Our statistical evaluation also indicates that gender influences patient subjective outcomes, with females experiencing less favorable results.

Webb et al. has found that the slope of the lateral tibial plateau may be a more sensitive risk factor for ACL injuries than the medial tibial plateau [39]. Prior studies have investigated the link between the lateral tibial slope (LTS) and the risk of graft failure after anterior cruciate ligament reconstruction (ACLR) [26, 27, 40–42]. These studies have suggested a range of LTS cutoff values to distinguish between groups at higher risk for graft failure and those at lower risk [26, 27, 43, 44]. However, the conclusions drawn from these studies vary, a discrepancy that can be attributed to the different methods employed in measuring LTS. In a 2019 case-control study, Grassi et al. investigated 43 patients who experienced graft failure after primary anterior cruciate ligament reconstruction (ACLR) [27]. They identified a lateral tibial slope (LTS) cutoff point of 7.4 degrees as a potential indicator of increased risk for graft failure. However, the specific surgical techniques used in these ACLR procedures were not detailed in their study. Additionally, it’s important to note that Grassi et al. employed a different methodology for measuring tibial slopes on MRI scans, which could influence the outcomes and comparability of their results. In a separate study, Cooper et al. focused on a larger cohort, enrolling 634 patients who had undergone primary single-bundle ACLR, including 317 individuals who required revision surgery and 317 who did not [28]. In this study, MRI scan measurements were performed using the technique described by Hashemi et al. One limitation of the Cooper et al. study was the lack of uniformity in graft types and femoral fixation methods used in the ACLR procedures, which could potentially affect the generalizability and interpretation of their findings. In our study, we adopted the measurement technique as described by Hashemi et al. for assessing the lateral and medial tibial slopes on MRI scans [45]. This approach allowed for a standardized and replicable method of evaluation, crucial for the reliability of our findings. Additionally, to maintain consistency in surgical variables, all patients in this study underwent single bundle anterior cruciate ligament reconstruction (ACLR) using the same graft type — hamstring tendon grafts. This consistency in both the measurement technique and surgical approach enhances the comparability of our results and provides a more controlled framework for analyzing the impact of tibial slope on post-operation ACLR subjective outcomes.

We found that an increased LTS was associated with poorer postoperative patient-reported outcome measures (PROMs), including Lysholm Knee Score, the UCLA Activity Score, the IKDC Score, and the Tegner Activity Score. This negative correlation indicates that a higher LTS may be predictive of less satisfactory outcomes following ACL reconstruction. Our findings are consistent with previous research indicating a relationship between higher LTS and increased risk of ACL injuries. Several biomechanical studies suggested that a higher LTS could increase anterior tibial translation, thereby increasing strain on the ACL. However, no previous studies had emphasized on the effect of increased tibial slope towards patient subjective outcomes. Our results suggest that the increased strain resulting from higher LTS could indeed impact the postoperative recovery and subjective outcomes following ACL reconstruction. This insight adds to our understanding of the complex interplay between biomechanical factors and patient-reported outcomes in the context of ACL injuries and reconstruction surgery.

While some studies suggest that double-bundle ACL reconstruction restores greater knee stability with respect to the antero-posterior and rotational stability than a single-bundle reconstruction, others have indicated that a single-bundle ACL reconstruction is sufficient to restore normal knee dynamic function [46, 47]. Our study recognizes the widely varied opinions and results between the difference in prognosis in single and double bundle ACL reconstruction. To provide a more targeted and controlled analysis, our research exclusively included patients who underwent single-bundle ACL reconstruction. This focused approach enables us to specifically investigate the prognosis and outcomes associated with this technique, thereby contributing valuable insights. By isolating this variable, our study aims to offer a clearer understanding of the correlation of LTS and subjective outcomes following single-bundle reconstruction.

Regarding the prognosis following anterior cruciate ligament (ACL) reconstruction between sexes, previous studies have demonstrated poorer outcomes in female patients compared to their male counterparts [48–50]. Females have inferior outcomes in instrumented laxity, revision rate, and activity scale after ACL reconstruction compared to males, but both sexes show comparable outcomes in other tests including anterior drawer test, Lachman test, pivot-shift test, timed single-legged hop test, single-legged hop test, quadriceps testing, hamstring testing, extension loss, flexion loss, development of cyclops lesion, and International Knee Documentation Committee (IKDC) knee examination score [48]. Some studies, however, found no difference in prognosis between sexes after ACL reconstruction surgery [51, 52]. Our study provides further evidence to support the gender disparity in ACL reconstruction results. In our cohort, female patients exhibited less favorable long-term PROMs and a statistically significant steeper lateral tibial slope (LTS), both of which are noteworthy factors contributing to the overall prognosis.

Furthermore, the established cut-off value of LTS in our study was 8.35 degrees, which was the point of distinction between patients with “Good” and “Fair” Lysholm scores. Previous studies have established varying cut-off values distinguishing better and inferior outcomes [26]. Webb et al. concluded that a posterior tibial slope (PTS) of above 12° had the most pronounced risk for ACL graft failure [39]. *PTS is the angle between the tibial anatomical axis and the tibial plateau tangent. This measurement is indicative of the tilt of the tibial plateau, which plays an important role in the biomechanics of the knee.* Grassi et al. established a cut-off value of 7.4° as an indicator for graft failure [27]. Gupta et al. found that an increased risk of graft failure was most evident with a posterior tibial slope (PTS) ≥ 10°, while Jaecker et al. observed similar findings for a lateral tibial posterior slope (LTPS) ≥ 10° [11, 53]. It is important to note that these studies exhibit variations to their methodologies, including patient selection, measurement techniques, and analysis, which can influence the interpretation and comparability of their results. Our study was designed to control for various variables, including hamstring grafts, single bundle surgical techniques, and the Hashemi measurement method, to ensure the reliability of our findings. However, our established cut-off value of LTS should nonetheless be interpreted with caution as LTS can still vary significantly among individuals and may be influenced by several factors, such as sex, age, and ethnicity. *Furthermore, given the multifaceted nature of ACL reconstruction outcomes, which can be affected by variables such as general joint laxity, lower limb alignment, and concurrent injuries to structures like the anterolateral ligament, our study’s focus on LTS alone might introduce a degree of bias in interpreting the results. Future revisions of this work could benefit from an in-depth examination of these additional determinants to present a more holistic view of factors influencing ACL reconstruction efficacy.*

### Limitation

Our study has several limitations. Firstly, the study was retrospective in nature, which could lead to potential selection biases that could affect the results. Additionally, the study was limited to a single institution, which could limit the generalizability of the results. Furthermore, arthrometer examination were not included in our study. GNRB arthrometer was not applied as part of our institution protocol for ACL injury patients until 2019. Finally, future study with larger sample sizes is needed to validate our findings and further explore the influence of LTS on ACL reconstruction outcomes.

## Conclusion

Our study suggests that a higher LTS may be associated with inferior subjective outcomes following single-bundle ACL reconstruction. Moreover, patients with a LTS of 8.35 and above are prone to exhibit worse subjective outcomes following single bundle ACL reconstruction surgery. This could be an important consideration during preoperative planning and patient counselling.

## Data Availability

The datasets used and/or analyzed during the current study are available from the corresponding author on reasonable request.

## Abbreviations

ACL: anterior cruciate ligament
LTS: lateral tibial slope
MTS: medial tibial slope
PROMS: Patient-reported outcome measures
IKDC: International Knee Documentation Committee Score
